# Reliability and agreement of manual and automated morphological radiographic hip measurements

**DOI:** 10.1016/j.ocarto.2024.100510

**Published:** 2024-08-14

**Authors:** F. Boel, N.S. Riedstra, J. Tang, D.F. Hanff, H. Ahedi, V. Arbabi, N.K. Arden, S.M.A. Bierma-Zeinstra, M.M.A. van Buuren, F.M. Cicuttini, T.F. Cootes, K. Crossley, D. Eygendaal, D.T. Felson, W.P. Gielis, J. Heerey, G. Jones, S. Kluzek, N.E. Lane, C. Lindner, J. Lynch, J. van Meurs, A.E. Nelson, A.B. Mosler, M.C. Nevitt, E.H. Oei, J. Runhaar, H. Weinans, R. Agricola

**Affiliations:** a Department of Orthopaedics and Sports Medicine, Erasmus Medical Center, Rotterdam, Zuid-Holland, the Netherlands; b Department of Radiology & Nuclear Medicine, Erasmus Medical Center, Rotterdam, Zuid-Holland, the Netherlands; c Institute for Medical Research, University of Tasmania Menzies, Hobart, Tasmania, Australia; d Department of Orthopedics, UMC Utrecht, Utrecht, the Netherlands; e Orthopaedic-Biomechanics Research Group, Department of Mechanical Engineering, Faculty of Engineering, University of Birjand, Birjand, Iran; f Department of Orthopaedics Rheumatology and Musculoskeletal Sciences, University of Oxford Nuffield, Oxford, Oxfordshire, UK; g Department of General Practice, Erasmus Medical Center, Rotterdam, Zuid-Holland, the Netherlands; h Department of Epidemiology and Preventative Medicine, Monash University, Melbourne, Victoria, Australia; i Centre for Imaging Sciences, The University of Manchester, Manchester, UK; j La Trobe Sport and Exercise Medicine Research Centre, La Trobe University School of Allied Health Human Services and Sport, Melbourne, Victoria, Australia; k Boston University School of Medicine, Boston, MA, USA; l Department of Medicine, University of California Davis School of Medicine, Sacramento, CA, USA; m Department of Epidemiology and Biostatistics, University of California San Francisco, San Francisco, CA, USA; n Thurston Arthritis Research Center, The University of North Carolina at Chapel Hill, Chapel Hill, NC, USA

**Keywords:** Hip joint, Hip shape, Automation, Morphology, Algorithm, Validation

## Abstract

**Objective:**

To determine the reliability and agreement of manual and automated morphological measurements, and agreement in morphological diagnoses.

**Methods:**

Thirty pelvic radiographs were randomly selected from the World COACH consortium. Manual and automated measurements of acetabular depth-width ratio (ADR), modified acetabular index (mAI), alpha angle (AA), Wiberg center edge angle (WCEA), lateral center edge angle (LCEA), extrusion index (EI), neck-shaft angle (NSA), and triangular index ratio (TIR) were performed. Bland-Altman plots and intraclass correlation coefficients (ICCs) were used to test reliability. Agreement in diagnosing acetabular dysplasia, pincer and cam morphology by manual and automated measurements was assessed using percentage agreement. Visualizations of all measurements were scored by a radiologist.

**Results:**

The Bland-Altman plots showed no to small mean differences between automated and manual measurements for all measurements except for ADR. Intraobserver ICCs of manual measurements ranged from 0.26 (95%-CI 0–0.57) for TIR to 0.95 (95%-CI 0.87–0.98) for LCEA. Interobserver ICCs of manual measurements ranged from 0.43 (95%-CI 0.10–0.68) for AA to 0.95 (95%-CI 0.86–0.98) for LCEA. Intermethod ICCs ranged from 0.46 (95%-CI 0.12–0.70) for AA to 0.89 (95%-CI 0.78–0.94) for LCEA. Radiographic diagnostic agreement ranged from 47% to 100% for the manual observers and 63%–96% for the automated method as assessed by the radiologist.

**Conclusion:**

The automated algorithm performed equally well compared to manual measurement by trained observers, attesting to its reliability and efficiency in rapidly computing morphological measurements. This validated method can aid clinical practice and accelerate hip osteoarthritis research.

## 1 Introduction

There is evidence that hip morphology is a leading contributing factor to the development of hip osteoarthritis (OA) [1]. Furthermore, studies have shown that specific hip morphologies, such as acetabular dysplasia (undercoverage of the femoral head by the acetabulum), pincer morphology (excessive coverage of the femoral head by the acetabulum) and cam morphology (aspherical femoral head) are associated with radiographic hip OA [[1], [2], [3], [4], [5], [6]].

In order to quantify hip morphology, morphological measurements can be performed on pelvic anteroposterior (AP) radiographs, which are inexpensive and routinely obtained in clinical practice. Manual morphological measurements, however, are time-consuming and can be unreliable when performed by different observers [7]. Additionally, a lack of consistency exists in the current definitions for some morphological measurements [8].

Automated morphological measurements could enhance reproducibility while facilitating rapid assessment of multiple measurements per radiograph. Automation, therefore, has the potential to aid clinical practice and allows for the quantification of hip morphology in large cohort studies. There are currently few open-access, publicly available algorithms, and those that are available are sometimes poorly described [[9], [10], [11]].

We aim to study the reliability and agreement of manual and our in-house developed, open-access, automated morphological hip measurements through quantitative and qualitative assessment of both methods. This ensures that results from future studies where this automated method is applied are clinically relevant. The secondary aim was to assess the agreement in making radiographic morphological diagnoses based on manual and automated measurements.

## 2 Methods

### 2.1 Participants

**Fig. 1:**
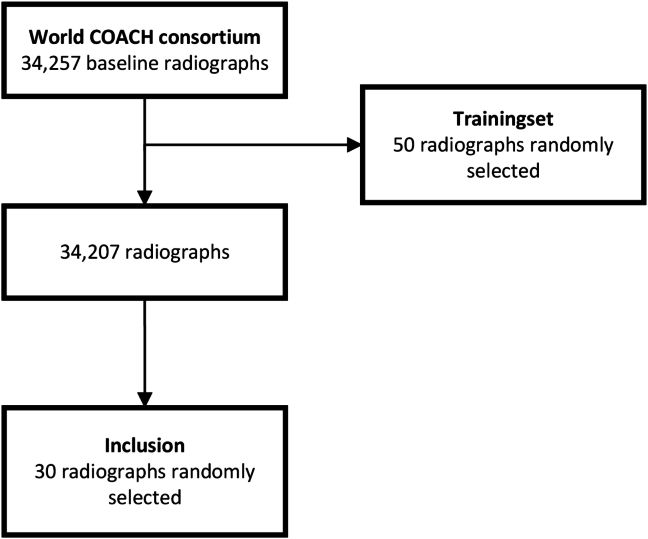
Flowchart of the radiograph selection.

The Worldwide Collaboration of OsteoArthritis prediCtion of the Hip (World COACH) consortium is a global collaboration of all prospective cohort studies with available sequential pelvic or hip imaging. The included cohorts are Cohort Hip and Cohort Knee, the Multi-center OSteoarthritis sTudy, the OsteoArthritis Initiative, the Rotterdam Study-I, the Rotterdam Study-II, the Rotterdam Study-III, the Chingford Study, the Johnston County Project, the Study of Osteoporotic Fractures, and the Tasmanian Older Adults Cohort. The World COACH consortium currently counts 37,732 participants aged 42–100 (mean 65.72 years) at baseline, and 71.33 % are female individuals. The consortium profile and protocol have previously been published in detail [12]. From the consortium, 30 baseline radiographs were selected proportionate to the cohort size in the consortium for qualitative and quantitative assessment of the manual and automated morphological measurements. A power analysis was performed assuming type I errors of 0.05, type II errors of 0.20, two replications, a minimally acceptable level of reliability of 0.75 and an expected level of reliability between 0.8 and 0.9, a minimum of 27 inclusions was needed. Therefore, we selected a total of 30 random radiographs for inclusion [13]. A flowchart of the radiograph selection is shown in Fig. 1. The baseline characteristics were: 18 females (60%), the mean age was 62.5 ​± ​8.6 years (range 47–78), and the mean BMI was 26.5 ​± ​3.9 ​kg/m2. All included hips had no definite RHOA as defined by Kellgren and Lawrence classification, modified Croft classification or modified OA score of 0 or 1.

### 2.2 Radiographs

The AP pelvic radiographs were obtained according to a protocol previously decided on by each cohort, and details on cohort-specific radiographic protocols can be found in the World COACH description paper [12]. Seven cohorts (CHECK, MOST, OAI, RS-I, RS-II, RS-III, TASOAC) contained weight-bearing AP pelvic radiographs. In contrast, three cohorts (the Chingford Study, JoCo, and SOF) contained supine AP pelvic radiographs.

### 2.3 Hip morphology and morphological measurements

**Fig. 2:**
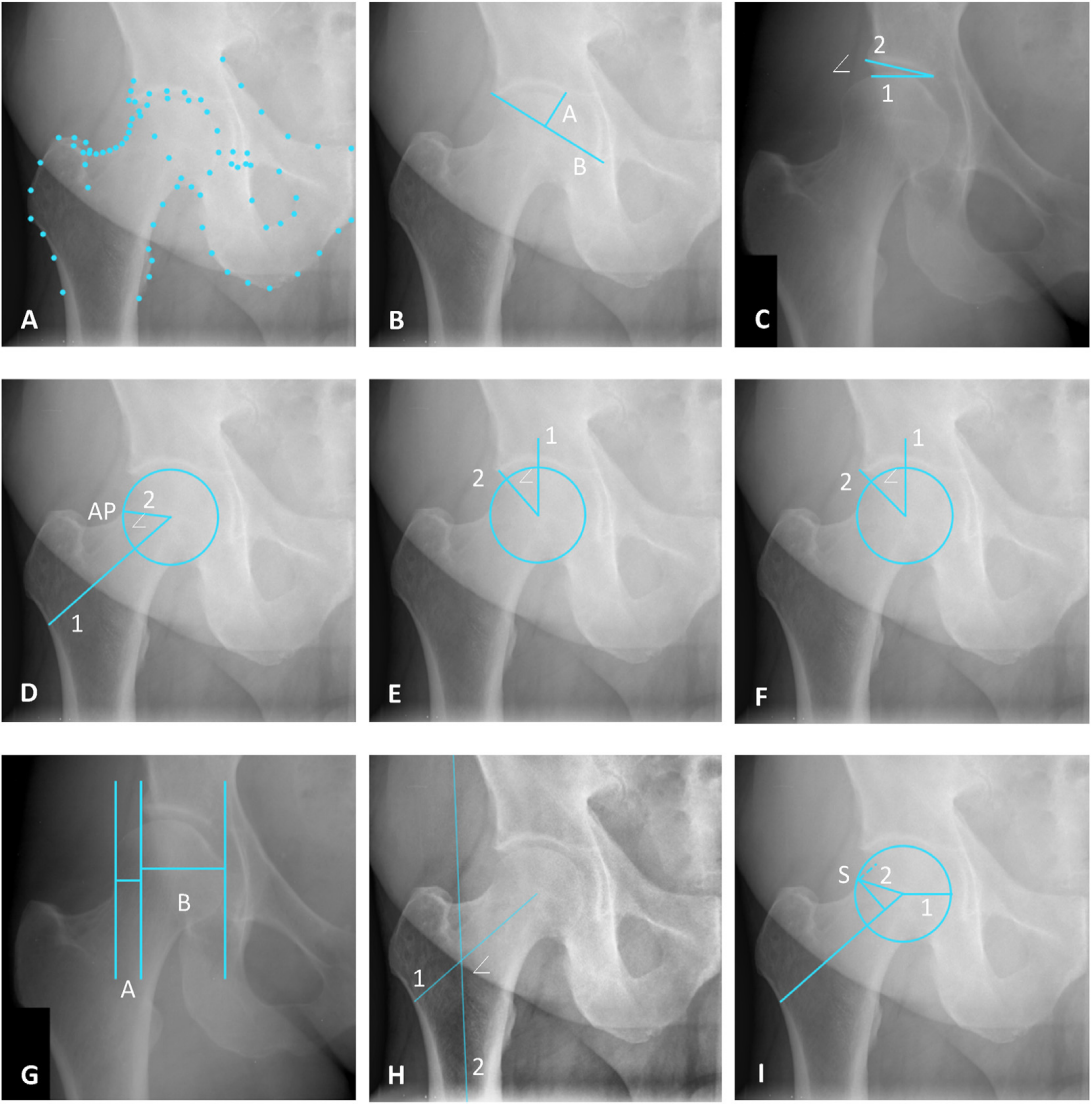
Definition of morphological measurements. **A:** Overview of the landmarks. **B: Acetabular depth-width ratio (ADR)** – the ratio between the acetabular depth (line A) measured from the most medial point of the acetabular sourcil to line B, and the acetabular width (line B) measured from the most lateral bony edge of the acetabulum to the most caudal point of the teardrop, ADR ​= ​A/B∗1000. **C: The mAI** – The angle between the horizontal reference line of the pelvis (line 1) and the line between the most lateral bony edge of the acetabulum and the most medial point of the acetabular sourcil (line 2). **D: The AA** – the angle between the femoral head-neck axis (line 1) and line 2 connecting the femoral head center and alpha point (AP), where the contour of the femoral head-neck junction leaves the best-fitting circle around the femoral head. **E: The WCEA** – The angle between line 1, a vertical line through the femoral head center perpendicular to the HRLP, and line 2 connecting the most lateral point of the acetabular sourcil and the femoral head center. **F: The LCEA** – The angle between line 1, a vertical line through the femoral head center perpendicular to the HRLP, and line 2 connecting the most lateral bony edge of the acetabulum and the femoral head center. **G: the EI** –EI ​= ​A/(A ​+ ​B)∗100%, where A is the distance between the most lateral point of the femoral head and the most lateral bony edge of the acetabulum, and B is the distance between the most lateral bony point of the acetabulum and the most medial point of the femoral head. **H: The NSA** – the angle between the femoral head-neck axis (line 1) and the longitudinal axis of the femoral shaft (line 2). **I: The TIR** – The ratio between the radius of the best-fitting circle around the femoral head (line 1) and the distance between the femoral head center and point S on the femoral head-neck junction at 0.5r along the femoral head-neck axis (line 2).

Morphological measures used in this manuscript to determine acetabular dysplasia include the acetabular depth-width ratio (ADR), the modified acetabular index (mAI), the Wiberg center edge angle (WCEA), and the extrusion index (EI) [[14], [15], [16]]. The lateral center edge (LCEA) angle determined pincer morphology [[17], [18], [19]]. Cam morphology was defined by the alpha angle (AA) and the triangular index ratio (TIR) [4,20,21]. The neck-shaft angle (NSA) is used to determine coxa valga and vara [22] All measurements are shown in Fig. 2 and are explained in detail elsewhere [23]; a brief overview, including radiological thresholds for radiographic diagnosis, is provided below.

#### 2.3.1 Acetabular depth-width ratio

The acetabular depth-width ratio (ADR) quantifies the depth of the acetabulum. The acetabular width was defined by a line from the lateral bony edge of the acetabulum to the pelvic teardrop to measure the acetabular opening. Next, the acetabular depth was defined by a line perpendicular to the acetabular width, extending from the most medial point of the sourcil (Fig. 2B). The ADR is the depth ratio to the width multiplied by 1000. Acetabular dysplasia is diagnosed by an ADR ≤250 [24].

#### 2.3.2 Modified Acetabular Index

The mAI measures the acetabular roof's inclination. The original acetabular index is applied to hips with an open triradiate cartilage; a modified version was created to obtain this measurement in adults. The mAI measures the angle between the line from the medial sourcil to the lateral bony edge of the acetabulum and the horizontal reference line of the pelvis (Fig. 2C). Acetabular dysplasia is defined by mAI ≥13°, acetabular overcoverage is defined by mAI ≤3° [24,25].

#### 2.3.3 Wiberg Center Edge Angle

The degrees of weight-bearing coverage of the femoral head by the acetabulum is measured by the WCEA [24]. The WCEA is formed by a vertical line through the center of the femoral head, perpendicular to the horizontal reference line of the pelvis, and a second line from the center of the femoral head to the most lateral weight-bearing part of the sourcil (Fig. 2E). Although the threshold has been debated, acetabular dysplasia is generally defined by a WCEA ≤25° in prospective studies [1,19,26,27].

#### 2.3.4 Lateral Center Edge Angle

The degrees of bony coverage of the femoral head by the acetabulum is measured by the LCEA [1,4,28].The LCEA is formed by a vertical line through the center of the femoral head, perpendicular to the horizontal reference line of the pelvis, and a second line from the center of the femoral head to the most lateral bony part of the acetabulum (Fig. 2F). Pincer morphology is generally defined by an LCEA ≥40° in prospective studies [1,17].

#### 2.3.5 Extrusion Index

The EI quantifies bony femoral head coverage by the acetabulum. The EI is obtained by dividing the horizontal distance of the lateral uncovered femoral head by the total width of the femoral head and multiplying that by 100 to express it as a percentage (Fig. 2G). Acetabular dysplasia is defined by an EI ≥ 25% [25].

#### 2.3.6 Alpha Angle

The AA is the most commonly used measurement to define cam morphology and quantify the sphericity of the femoral head-neck junction. The AA is constructed by two lines, one from the femoral head center through the middle of the femoral neck, the femoral head-neck axis, and a second line from the center of the femoral head through the point where the contour of the femoral head-neck junction extends from the best fitting circle around the femoral head (Fig. 2D) [29]. An AA ≥ 60° threshold is commonly used in literature to define cam morphology [20].

#### 2.3.7 Triangular Index Ratio

The TIR measures femoral asphericity and defines cam morphology. Compared to the AA, the TIR is measured at a specific point on the femoral head-neck junction. It is the ratio between the radius of the best-fitting circle around the femoral head and the distance between the femoral head center and the femoral head-neck junction at 0.5r along the head-neck axis (Fig. 2I). When, for instance, the resultant distance at 0.5r along the axis of the femoral neck at the head-neck junction exceeds the radius of the femoral head, this indicates that, the femoral head is aspherical, possibly indicating the presence of cam morphology [21].

#### 2.3.8 Neck-shaft Angle

The NSA is the angle between the longitudinal axis of the femoral shaft and the femoral head-neck axis (Fig. 2H). It has been hypothesized that hips with a more varus neck orientation experience increased subchondral bone stress and, therefore, increased risk of degeneration in individuals with cam morphology [30]. Conversely, a relative increase in femoral neck shaft angle combined with acetabular undercoverage also leads to RHOA [30]. Coxa valga is generally defined by NSA> 140°, and coxa vara by NSA< 120° [31].

### 2.4 Automated morphological measurements

The bony outline of the proximal femur and acetabulum were annotated automatically on all AP pelvic radiographs with a landmarks (Fig. 2A) (BoneFinder® software (www.bone-finder.com; The University of Manchester, UK) [32]. The protocol for the 80 landmarks used in this automated hip shape annotation can be found in supplementary material 1. The landmarks were used to automatically derive the hip morphology measurements using in-house-built Python-based software [23]. This software is a pipeline to automatically determine radiographic measurements based on radiographic landmarks. The radiographic measurements are performed in accordance to the definitions provided in this manuscript [23]. To assess the impact of automated landmark placement on the morphological measurements, a second set of landmarks was created on the same set of radiographs where all landmarks were manually assessed and adjusted, if necessary, after which the morphological measurements were derived again.

### 2.5 Manual morphological measurements

Two researchers (JT and NSR) were trained in performing manual assessment of all previously described morphological measurements. A random set of 50 radiographs from the World COACH consortium was used to train the researchers. Radiographs were selected at random from the consortium such that the number of radiographs chosen from each cohort was proportional to the total number of radiographs available in that cohort. After all measurements were performed on all 50 radiographs by both researchers, measurements were compared under supervision of an experienced orthopedic surgeon (RA), and inconsistencies were discussed. This was repeated 3 times with the same radiographs until both researchers were proficient in performing measurements. Next, the two trained researchers (JT and NSR) performed on the 30 randomly selected radiographs from the World COACH consortium, with the same proportionality as previously mentioned. Information on whether the hips had morphological variations, hip OA, or clinical symptoms was blinded to all researchers. The measurements were repeated on the same radiographs approximately four weeks later. The radiographs were presented to the readers in a different random order each time. Measurements were performed using the DICOM viewer (Synedra View, Version 21.0.0, Synedra Information Technologies). All radiographs were presented in a blinded fashion and random order to the observers. The mean of the individual observers' first and second round of measurements was used for interobserver analyses. The mean of all four manual measurements was used as the reference standard to which the automated method was compared.

### 2.6 Agreement

The agreement within the two rounds of manual measurements for each observer and between observers, and between methods with regard to radiographic diagnoses solely based on morphological measurements of acetabular dysplasia, pincer and cam morphology, and coxa vara and valga was tested.

### 2.7 Qualitative assessment of morphological measurements

A musculoskeletal radiologist (DFH) visually inspected the second round of manual morphological measurements and the automated measurements based on the unadjusted landmarks and qualitatively rated the measurements as acceptable or unacceptable. “Acceptable” is if the radiologist would measure the same morphological measurements based on the landmark points. “Unacceptable” is if the radiologist would perform the measurements differently. This was done in order to ensure the automated measurements were correct from a clinical perspective of an MSK radiologist. In order to blind the radiologist to which method was used, Printscreens of the manual and automated measurements were visually presented in a way which made it impossible to distinguish between methods and in a random order. Printscreens were used because automated measurements were obtained in Python and manual measurements in Synedra Viewer, which would distinguish between methods. Additionally, this ensured that our reference standard of manual measurements were also approved by the MSK radiologist. An example of the ADR is shown in supplementary material 2. No additional information was disclosed about whether the measurements were performed manually or obtained by the automated method.

### 2.8 Statistical analysis

The agreement between the manual observers and the agreement between the automated and manual methods was visualized using Bland-Altman plots for each morphological measurement. In this study, in order to distunguish between random and systematic error, a mean difference larger than 2.5° was defined as a systematic error for mAI, AA, WCEA, LCEA and NSA. A mean difference larger than 1% of the measurement was defined as a systematic error for ADR, EI and TIR. These thresholds are based on expert agreement. Outliers identified by the Bland-Altman plots were visually inspected to analyze whether consistencies in measurement error occurred.

Intraclass correlation coefficients (ICCs) were used to test reliability and were reported with 95% confidence intervals (CI). Intraobserver reliability was tested with a 2-way mixed-effects model, single rater, absolute agreement ICC. Interobserver reliability between manual observers and between the automated determination of the measurements on the manually adjusted and unadjusted landmarks was tested with a 2-way random-effects model, single rater, absolute agreement ICC. Lastly, intermethod reliability between the mean of all manual and automated measurements on manually adjusted and unadjusted landmarks was tested with a 2-way mixed-effects model, single rater, absolute agreement ICC. ICCs were rated as poor (<0.50), moderate (0.50–0.75), good (0.76–0.90), or excellent (>0.90) [33].

The agreement within and between observers, and between methods with regard to radiographic diagnoses was tested using percentage agreement. Based on the qualitative rating of the measurements by the musculoskeletal radiologist, the percentage of acceptable measurements was determined for each morphological measurement by the two manual observers and the automated method, respectively. The percentage of acceptable measurements was rated as poor (<50%), moderate (50–70%), good (71–90%), or excellent (>90%).

Statistical analyses were performed using R statistical software (v4.1.0; R Core Team 2021). The ggplot2-package in R was used to create Bland-Altman plots. The irr-package in R was used to calculate the ICCs and the percentage agreement [34].

## 3 Results

All morphological measurements could automatically be performed in all 30 hips, except for NSA, which could not be performed on two images as too little of the femoral shaft was depicted on the radiograph.

### 3.1 Agreement

**Fig. 3:**
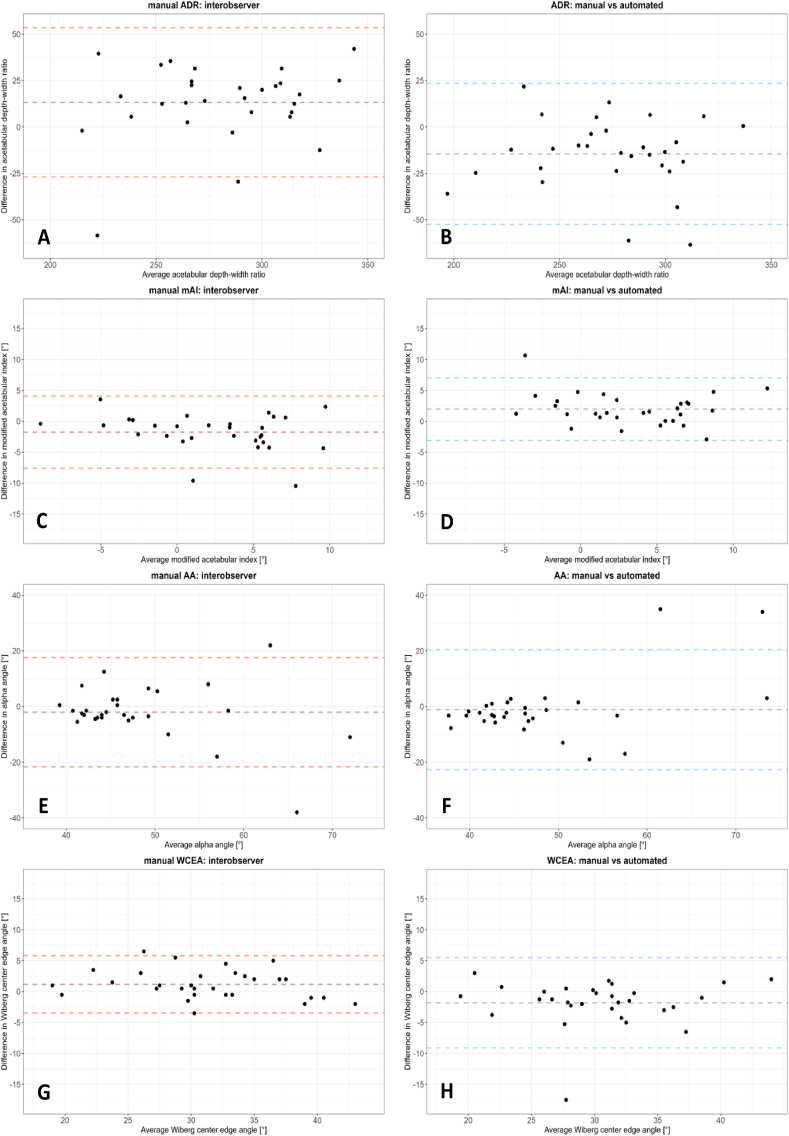

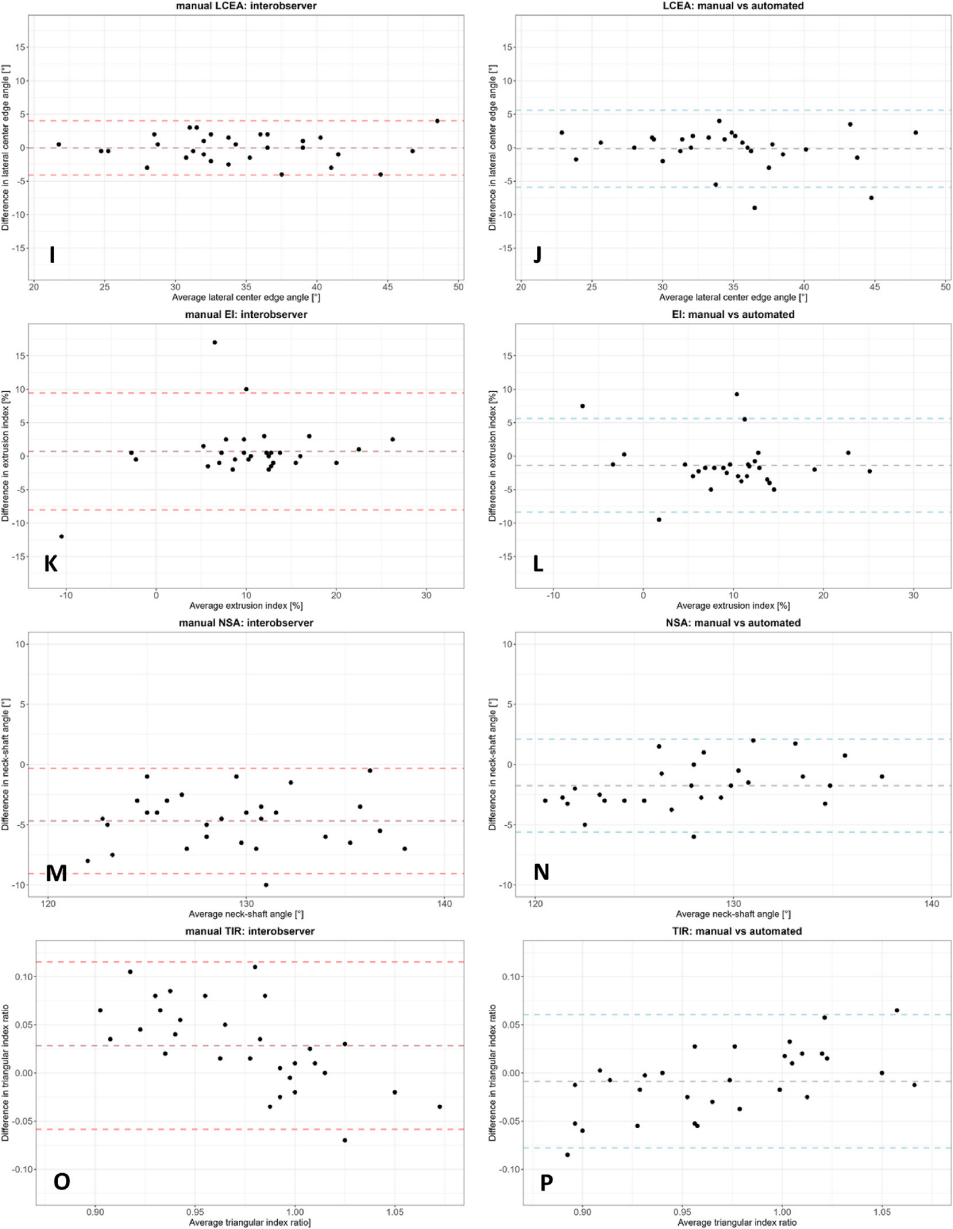
Bland-Altman plots of the morphological measurements. **A**: The acetabular depth-width ratio (ADR) – observer 1 vs observer 2. **B**: ADR – manual vs automated measurements based on unadjusted landmarks. **C**: The mAI – observer 1 vs observer 2. **D**: mAI – manual vs automated measurements based on unadjusted landmarks. **E**: The AA – observer 1 vs observer 2. **F**: AA – manual vs automated measurements based on unadjusted landmarks. **G**: The WCEA – observer 1 vs observer 2. **H**: WCEA – manual vs automated measurements based on unadjusted landmarks. **I**: The LCEA – observer 1 vs observer 2. **J**: LCEA – manual vs automated measurements based on unadjusted landmarks. **K**: The EI – observer 1 vs observer 2. **L**: EI – manual vs automated measurements based on unadjusted landmarks. **M**: The NSA – observer 1 vs observer 2. **N**: NSA – manual vs automated measurements based on unadjusted landmarks. **O**: The TIR – observer 1 vs observer 2. **P**: TIR – manual vs automated measurements based on unadjusted landmarks.

The Bland-Altman plots for agreement between the two observers and the agreement between the manual and automated measurements based on unadjusted landmarks are presented in Fig. 3, and the corresponding mean difference and limits of agreement are summarized in Table 1. The AA, WCEA, LCEA, mAI, and EI showed no to small mean differences between automated and manual measurements. However, both the interobserver and intermethod agreement of ADR and the interobserver NSA and TIR showed a bias. Observer 1 consistently measured ADR and TIR higher than observer 2, while the opposite was observed for ADR. When comparing the manual and automated ADR, the mean of the manual measurements was consistently higher than the automated measurement.

**Table 1:** Summary of mean interobserver and intermethod bias and limits of agreement of manual morphological measurements and manual vs automated morphological measurements based on the unadjusted landmarks.

Measurement	Manual		Manual vs Automated	
	Interobserver bias (mean)	Interobserver limits of agreement	Intermethod bias (mean)	Intermethod limits of agreement
Acetabular depth-width ratio	13	−27 to 53	−15	−52 to 13
Modified acetabular index [°]	−1.8	−7.6 to 4.1	2.0	−3.1 to 7.0
Alpha angle [°]	−2	−22 to 18	−1	−23 to 20
Wiberg center edge angle [°]	1	−3 to 6	−2	−9 to 5
Lateral center edge angle [°]	0	−4 to 4	0	−6 to 6
Extrusion index [%]	1	−8 to 9	−1	−8 to 5
Neck-shaft angle [°]	−5	−9 to 0	−2∗	−6 to 2∗
Triangular index ratio	0.028	−0.058 to 0.115	−0.009	−0.078 to 0.061

Bland-Altman interobserver and intermethod bias (mean difference) and limits of agreement, n=30. ∗Based on 28 hips.

The intermethod limits of agreement were mainly smaller or similar to the interobserver limits of agreement for all morphological measurements except for WCEA and LCEA.

### 3.2 Reliability

The intra- and interobserver and intermethod reliability defined by ICCs for all measurements are shown in Table 2. The intermethod reliability between the manual and automated measurements based on both the manually adjusted and unadjusted landmarks was comparable to or better than the interobserver reliability, except for WCEA in which case the manual measurements were more reliable. Additionally, we found that manually adjusted landmarks impacted the ADR and mAI most. This led to lower reliability between manually adjusted compared to unadjusted automated ADR and mAI measurements. These measurements are calculated based on only on few specific landmarks. Conversely, measurements that do not rely on few specific landmarks from the point set like AA, NSA and TIR, showed excellent reliability between the automated measurements performed using the adjusted vs unadjusted landmarks.

**Table 2:** Intra- and interobserver reliability between manual measurements by observer 1 and observer 2, interobserver reliability between adjusted and unadjusted landmarks and intermethod reliability between manual and automated morphological measurements.

	Manual			Automated	Manual vs automated	
	Observer 1	Observer 2	Observer 1 vs observer 2	Adjusted vs unadjusted landmarks	Unadjusted landmarks	Adjusted landmarks
Measurement	Intraobserver ICC (95% CI)	Intraobserver ICC (95% CI)	Interobserver ICC (95% CI)	Interobserver ICC (95% CI)	Intermethod ICC (95% CI)	Intermethod ICC (95% CI)
Acetabular depth-width ratio	0.67 (0.41–0.82)	0.89 (0.77–0.94)	0.79 (0.49–0.91)	0.70 (0.47–0.85)	0.78 (0.39–0.91)	0.80 (0.60–0.90)
Modified Acetabular Index	0.65 (0.36–0.82)	0.82 (0.61–0.91)	0.77 (0.48–0.89)	0.83 (0.67–0.91)	0.75 (0.34–0.90)	0.82 (0.30–0.94)
Alpha Angle	0.36 (0.01–0.63)	0.67 (0.42–0.83)	0.43 (0.10–0.68)	0.98 (0.97–0.99)	0.46 (0.12–0.70)	0.5 (0.17–0.73)
Wiberg center edge angle	0.87 (0.74–0.93)	0.94 (0.88–0.97)	0.91 (0.78–0.96)	0.94 (0.88–0.97)	0.77 (0.54–0.89)	0.88 (0.70–0.95)
Lateral center edge angle	0.89 (0.78–0.95)	0.95 (0.87–0.98)	0.95 (0.86–0.98)	0.91 (0.8–0.96)	0.89 (0.78–0.94)	0.95 (0.88–0.98)
Extrusion Index	0.74 (0.51–0.87)	0.80 (0.51–0.91)	0.83 (0.67–0.91)	0.94 (0.87–0.97)	0.86 (0.71–0.93)	0.88 (0.65–0.95)
Neck Shaft Angle	0.89 (0.78–0.94)	0.86 (0.73–0.93)	0.58 (0–0.87)	0.995 (0.989–0.998)^a^	0.86 (0.44–0.95)^a^	0.88 (0.51–0.96)^a^
Triangular Index Ratio	0.26 (0–0.57)	0.88 (0.76–0.94)	0.49 (0.12–0.73)	0.99 (0.98–0.996)	0.78 (0.59–0.89)	0.79 (0.61–0.89)

Intraclass correlation coefficients (ICC) of intra- and interobserver, and intermethod reliability of the morphological measurements. ICCs are presented with 95% confidence interval (CI). The mean of all four manual measurements was used as the reference standard for the intermethod measurements. Intraobserver reliability was tested with a 2-way mixed-effects model, single rater, absolute agreement ICC. Interobserver reliability between both manual observers, as well as between the automated determination on adjusted and unadjusted landmarks, was tested with a 2-way random-effects model, single rater, absolute agreement ICC. Intermethod reliability was tested with a 2-ways mixed-effects model, single rater, absolute agreement ICC. All ICCs were measured using 30 hips.

a ICCs measured using 28 hips. Interpretation: poor (<0.50), moderate (0.50–0.75), good (0.76–0.90), or excellent (>0.90).

### 3.3 Radiographic diagnostic agreement

Percentage agreement in radiographic diagnosis based on morphological measurements is summarized in Table 3. The intermethod radiographic diagnostic agreement was better than or similar to the interobserver radiographic diagnostic agreement. Except for the radiographic diagnostic agreement of dysplasia based on mAI of the manual versus automated measurements based on the manually adjusted landmarks.

**Table 3:** Prevalence and diagnostic intraobserver and interobserver agreement between observer 1 and observer 2, interobserver agreement between adjusted and unadjusted automated measurements, and intermethod agreement.

	Manual			Automated	Manual vs automated			
	Observer 1	Observer 2	Observer 1 vs observer 2	Adjusted vs unadjusted landmarks	Reference standard	Unadjusted landmarks	Unadjusted landmarks	Adjusted landmarks
Measurement	Intraobserver percent agreement	Intraobserver percent agreement	Interobserver percent agreement	Interobserver percent agreement	Prevalence	Prevalence	Intermethod percent agreement	Intermethod percent agreement
Acetabular depth-width ratio ≤250	90.0	83.3	86.7	93.3	16.7%	26.7%	90.0	90.0
Modified acetabular Index ≥13°	96.7	96.7	96.7	90.0	0%	3.3%	96.7	86.7
Alpha Angle ≥60°	90.0	90.0	86.7	96.7	10.0%	10.0%	86.7	83.3
Wiberg center edge angle ≤25°	100.0	100.0	93.3	90.0	13.3%	23.3%	96.7	93.3
Lateral center edge angle ≥40°	90	93.3	93.3	96.7	20.0%	16.7%	96.7	100
Extrusion Index ≥25%	96.7	96.7	100	100	0%	0%	100	100
Neck Shaft Angle <120° & >140°	90	96.7	90	96.7	<120°: 0% >140°: 0%	<120°: 3.3% >140°: 0%	96.7	100

The reference standard consists of the mean of all manual measurements. Intermethod percent agreement was determined using the reference standard. n ​= ​30.

### 3.4 Qualitative assessment

The results of the qualitative assessment as performed by the MSK radiologist are presented in Table 4. The majority of automated measurements were deemed acceptable by the musculoskeletal radiologist. The percentage of acceptable measurements was moderate to excellent for all measurements, except for the EI measurements by observer 2.

**Table 4:** The qualitative assessment of the morphological measurements.

Measurement	Manual		Automated
	Observer 1	Observer 2	Unadjusted landmarks
Acetabular depth-width ratio	77	80	73
Modified Acetabular Index	70	53	70
Alpha Angle	93	90	77
Wiberg center edge angle	73	80	63
Lateral center edge angle	70	90	80
Extrusion Index	53	47	63
Neck Shaft Angle	93	100	96^a^
Triangular Index Ratio	63	100	73

Percentage of acceptable measurements. Qualitative assessment was performed on 30 hips.

a Based on only 28 hips. Interpretation: poor (<50%), moderate (50–70%), good (71–90%), or excellent (>90%).

## 4 Discussion

This study investigated the agreement and reliability of manual and automated morphological measurements including ADR, mAI, AA, WCEA, LCEA, EI, NSA, and TIR on AP pelvic radiographs. The presented algorithm performed equally well compared to current best practice of manual measurement by trained readers, attesting to its reliability and efficiency in rapidly computing radiological measurements on an AP pelvic radiograph.

The reported intra- and interobserver reliability of morphological measurements varies in literature. The reported ICCs in the present study were compared to the reliability of various morphological measurements in literature. The ICCs reported in literature for the Wiberg and lateral CEA (ICC ​= ​0.7 (95% CI 0.58–0.86) to 0.98 (CI 0.97–0.99) [33,[35], [36], [37], [38]] the NSA (ICC ​= ​0.58 (0.31–0.76) to 0.98 (0.95–0.99) [38]), the mAI (or Tönnis angle) (ICC ​= ​0.71 (95% CI 0.45–0.83) to 0.92 (95% CI 0.85–0.95) [33,35,39]), the EI (ICC ​= ​0.68 (0.57–0.79) to 0.98 (no CI reported) [33,[35], [36], [37]] and the ADR (ICC ​= ​0.62 to 0.84 [37,39,40] are similar to the ICCs found in our study. The reported reliability in literature for the AA (ICC ​= ​0.78 (95% CI 0.61–0.87) to 0.99 (no CI reported) [[41], [42], [43]]) is higher than observed in the present study. No reliability has been reported for the TIR, although one study did report on the triangular index height in 10 individuals (***κ*** ​= ​0.74–0.78) [33].

In terms of reliability and agreement in the current study, the AA showed the worst reliability in the manual method between and within observers, as well as in terms of intermethod reliability. The AA also showed large limits of agreement in the Bland-Altman plots and erratic behavior in the higher AA values (representing cam hips). These results are likely caused by small differences in femoral head circle fit, which may cause large measurement variation due to movement of the alpha point (Fig. 3). Faber et al. showed similar outliers and erratic behavior within the Bland-Altman analysis when comparing manual and automated AA measurements [44]. Similar results, although less extreme, were found for TIR, as expected since this measurement is also largely dependent on the circle fit. However, the erratic behavior observed in the AA Bland-Altman plots in hips with cam morphology is absent in the TIR Bland-Altman plots. This may be caused by the fact that compared to the location of the alpha point, the location of point S (Fig. 2I) is less influenced by the best-fitting circle around the femoral head.

ADR and mAI are two measurements which are calculated based on only two to three landmarks and, therefore highly dependent on correct landmarks recognition and placement. This is reflected in similar reliability and limits of agreement for the intra- and interobserver, and intermethod comparisons. The outliers in these measurements were all caused by different landmarks recognition and placement of both the most lateral bony edge of the acetabulum and the most medial point of the weight-bearing sourcil. Additionally, we found that the mean of the manual measurements by the trained researchers was consistently higher than the automated measurement, implying that we may under diagnose acetabular dysplasia based on manual ADR measurements. Alternatively, it may also be the case that the medial point of the ADR on the sourcil is difficult to identify for the automated measurement. This may also influence the automated ADR.

The correct identification of the most lateral bony edge of the acetabulum also influenced the LCEA and EI measurements. The reliability was good to excellent for all analyses, and the limits of agreement were similar between the interobserver and intermethod analyses.

The WCEA, as determined using the automated method, was slightly worse than the LCEA when comparing the automated method to manual measurements. This is likely due to more difficult assessment of the sourcil, than the more distinct lateral bony acetabular rim. This is also observed in literature with higher reliability for LCEA reported compared to WCEA [[32], [33], [34], [35], [36], [37]]. Overall, this landmark needed more adjustment than the most lateral bony part of the acetabulum during the manual assessment of landmarks placement. This was reflected in the higher reliability of the manual versus automated measurement when the WCEA was performed based on the manually adjusted landmarks.

The majority of manual measurements were deemed acceptable by the musculoskeletal radiologist. This implies that the reported manual measurement ICCs represent clinically acceptable reliability. In terms of automated measurements, we can conclude that the automated ADR, mAI, AA, LCEA, NSA and TIR measurements are valid in a clinical setting and can be applied to establish radiographic morphological hip diagnoses. According to our study, performance of manual as well as automated EI measurements does not reach the threshold for good agreement. We hypothesize that in case of less sphericity of the femoral head, the identification of the most lateral point of the femoral head becomes difficult leading to unreliability in the measurement. As there are other measurements that quantify acetabular coverage, these may be more appropriate in a clinical setting to study hip morphology.

Using automated morphological measurements may advance research and have important clinical implications. First, automated measurements may improve accuracy and consistency in morphological measurements reported in literature. Measurement variability and bias could be reduced dramatically if all measurements are performed uniformly, allowing for comparison of results across studies. This holds especially true in terms of the femoral head circle fit, which is essential in many morphological measurements. The present automated method is published open-access [23], which promotes collaboration in future hip (OA) studies. While the method is still reliant on correct landmark identification, this was also automated to achieve more consistency and speed. This method can be applied in future studies to study whether these measurements are associated with clinical outcomes such as symptomatic hip OA. The automated method was tested on supine and standing pelvic radiographs from various cohorts in the World COACH consortium, potentially making the results more generalizable to a larger population. Furthermore, the automated method can improve efficiency by accommodating the collection of large amounts of morphological data. This will allow researchers to carry out studies with increased statistical power, advancing our understanding of hip morphology as a risk factor for hip OA.

No gold standard is available for these morphological measurements, so we extensively trained researchers to obtain measurements which could be used as a reference standard. We found order to ensure that these measurements resemble clinical practice, an MSK radiologist visually inspected all manual and automated measurements. Secondly, it should be kept in mind that this study includes a rather small set of 30 hips. A larger dataset would likely show increased variation in hip morphology and therefore provide a more robust assessment of the described methods. Furthermore, as the participants from the World COACH consortium are either from the general population or from a population selected based on having symptoms or risk factors for hip OA, the hips are a representation of the normal population. Therefore, gross bony deformations as seen in hospital populations are underrepresented in the world COACH consortium and results from the automated measures should be validated in this population first. All thresholds used to define radiographic morphological diagnoses are based on literature, but what the “right” threshold is remains unknown [45].

With regards to the qualitative assessment, the radiologist evaluated printscreens of measurements, which made it impossible to adjust contrast setting on the images as preferred by the radiologist. As a result of this, the measurements that were impossible to visually inspect were labeled as unacceptable, although in reality they may have been correct. This issue may be avoided in the future by using DICOM images on PACS viewer rather than printscreens of radiographs. Another limitation of this study is that all morphological measurements were performed on AP pelvic radiographs although it is known that some morphological diagnoses require additional radiographic views to assess hip morphology [19,25,33,37]. Furthermore, acetabular morphology is influenced by pelvic orientation, which can vary significantly in terms of tilt [46]. This provides a future opportunity to also develop automated measurements in various radiographic views.

In conclusion, automated morphological measurements are a reliable and reproducible method to quantify the ADR, WCEA, LCEA mAI, TIR, EI and NSA. This method makes morphological hip measurements viable in large population studies, as it enables reliable analysis of large amounts of data. Additionally, it may be a useful tool in clinical practice, as it reduces reader bias and the landmarks allow for insightful measurements. Access to fast, externally validated, reliable methods to quantify hip morphology may aid in the quest for modifiable risk factors for hip OA in future studies.

## Funding

CL is funded by a Sir Henry Dale Fellowship jointly funded by the Wellcome Trust and the Royal Society (223267/Z/21/Z). For the purposes of open access, the authors have applied a CC BY public copyright licence to any Author Accepted Manuscript version arising from this submission.

The World COACH consortium has been funded through research grants by the Dutch Arthritis Society (grant no. 18-2-203 and 21-1-205), the Dutch Research Council (NWO Veni grant scheme no. 09150161910071), and Erasmus MC, University Medical Center Rotterdam (Erasmus MC Fellowship).

## Conflict of Interest

GJ reports personal fees from Novartis outside the submitted work. SBZ reports consulting fees from Pfizer Infirst Healthcare and personal fees for being a Deputy Editor for Osteoarthritis and Cartilage outside the submitted work. CL and TC report a patent for an image processing apparatus and method for fitting a deformable shape model to an image using random forest regression voting. CL reports licensing royalties for this patent from Optasia Medical outside the submitted work. AN is an associate editor for Osteoarthritis and Cartilage and is on the OARSI Board of Directors outside the submitted work. AM is on the Editorial Board for the British Journal of Sports Medicine and the Journal of Science and Medicine in Sport outside the submitted work. HW reports being a minority shareholder of Uplanner BV and Replasia BV outside the submitted work.

## References

[1] Casartelli N.C., Maffiuletti N.A., Valenzuela P.L., Grassi A., Ferrari E., van Buuren M.M.A. (2021). Is hip morphology a risk factor for developing hip osteoarthritis? A systematic review with meta-analysis. Osteoarthritis Cartilage.

[2] Thomas G.E., Kiran A., Batra R.N., Hart D., Spector T., Taylor A. (2012). The association between hip morphology and end-stage osteoarthritis at 12-year follow up. Osteoarthritis Cartilage.

[3] Hoch A., Schenk P., Jentzsch T., Rahm S., Zingg P.O. (2021). FAI morphology increases the risk for osteoarthritis in young people with a minimum follow-up of 25 years. Arch. Orthop. Trauma Surg..

[4] Van Klij P., Heerey J., Waarsing J.H., Agricola R. (2018). The prevalence of cam and pincer morphology and its association with development of hip osteoarthritis. J. Orthop. Sports Phys. Ther..

[5] Beck M., Kalhor M., Leunig M., Ganz R. (2005). Hip morphology influences the pattern of damage to the acetabular cartilage: femoroacetabular impingement as a cause of early osteoarthritis of the hip. J. Bone Joint Surg..

[6] Harris-Hayes M., Royer N.K. (2011). Relationship of acetabular dysplasia and femoroacetabular impingement to hip osteoarthritis: a focused review. PM&R.

[7] Hanson J.A., Kapron A.L., Swenson K.M., Maak T.G., Peters C.L., Aoki S.K. (2015). Discrepancies in measuring acetabular coverage: revisiting the anterior and lateral center edge angles. J Hip Preserv Surg.

[8] Griffin D.R., Dickenson E.J., O'donnell J., Awan T., Beck M., Clohisy J.C. (2016). The Warwick Agreement on femoroacetabular impingement syndrome (FAI syndrome): an international consensus statement. Br. J. Sports Med..

[9] Schwarz G.M., Simon S., Mitterer J.A., Huber S., Frank B.J., Aichmair A. (2023). Can an artificial intelligence powered software reliably assess pelvic radiographs?. Int. Orthop..

[10] Faber B.G., Ebsim R., Saunders F.R., Frysz M., Smith G.D., Cootes T. (2021). Deriving alpha angle from anterior-posterior dual-energy x-ray absorptiometry scans: an automated and validated approach. Wellcome open research.

[11] Archer H., Reine S., Alshaikhsalama A., Wells J., Kohli A., Vazquez L. (2022). Artificial intelligence-generated hip radiological measurements are fast and adequate for reliable assessment of hip dysplasia: an external validation study. Bone & Joint Open.

[12] van Buuren M.M.A., Riedstra N.S., van den Berg M.A., Boel F., Ahedi H., Arbabi V., Arden N.K., Bierma-Zeinstra S.M.A., Boer C.G., Cicuttini F.M., Cootes T.F., Crossley K.M., Felson D.T., Gielis W.P., Heerey J.J., Jones G., Kluzek S., Lane N.E., Lindner C., Lynch J.A., van Meurs J.B.J., Mosler A., Nelson A.E., Nevitt M.C., Oei E.H.G., Runhaar J., Tang J., Weinans H. (2024). Cohort profile: Worldwide Collaboration on OsteoArthritis prediCtion for the Hip (World COACH); an international consortium of prospective cohort studies with individual participant data on hip osteoarthritis. BMJ Open.

[13] Walter S.D., Eliasziw M., Donner A. (1998 Jan 15). Sample size and optimal designs for reliability studies. Stat. Med..

[14] Engesæter I.Ø., Laborie L.B., Lehmann T.G., Sera F., Fevang J., Pedersen D. (2012 Jul). Radiological findings for hip dysplasia at skeletal maturity. Validation of digital and manual measurement techniques. Skeletal Radiol..

[15] Umer M., Thambyah A., Tan W., De S.D. (2006). Acetabular morphometry for determining hip dysplasia in the Singaporean population. J. Orthop. Surg..

[16] Engesæter I.Ø., Laborie L.B., Lehmann T.G., Fevang J.M., Lie S.A., Engesæter L.B. (2013). Prevalence of radiographic findings associated with hip dysplasia in a population-based cohort of 2081 19-year-old Norwegians. The Bone & Joint Journal.

[17] Agricola R., Heijboer M.P., Roze R.H., Reijman M., Bierma-Zeinstra S.M.A., Verhaar J.A.N. (2013). Pincer deformity does not lead to osteoarthritis of the hip whereas acetabular dysplasia does: acetabular coverage and development of osteoarthritis in a nationwide prospective cohort study (CHECK). Osteoarthritis Cartilage.

[18] Faber B.G., Ebsim R., Saunders F.R., Frysz M., Gregory J.S., Aspden R.M. (2021). Cam morphology but neither acetabular dysplasia nor pincer morphology is associated with osteophytosis throughout the hip: findings from a cross-sectional study in UK Biobank. Osteoarthritis Cartilage.

[19] Saberi Hosnijeh F., Zuiderwijk M.E., Versteeg M., Smeele H.T., Hofman A., Uitterlinden A.G. (2017 Jan). Cam deformity and acetabular dysplasia as risk factors for hip osteoarthritis. Arthritis Rheumatol..

[20] van Klij P., Reiman M.P., Waarsing J.H., Reijman M., Bramer W.M., Verhaar J.A.N. (2020 Aug 10). Classifying cam morphology by the alpha angle: a systematic review on threshold values. Orthop J Sports Med.

[21] Gosvig K.K., Jacobsen S., Palm H., Sonne-Holm S., Magnusson E. (2007). A new radiological index for assessing asphericity of the femoral head in cam impingement. The Journal of Bone & Joint Surgery British.

[22] Ramkumar P.N., Karnuta J.M., Haeberle H.S., Sullivan S.W., Nawabi D.H., Ranawat A.S. (2020). Radiographic indices are not predictive of clinical outcomes among 1735 patients indicated for hip arthroscopic surgery: a machine learning analysis. Am. J. Sports Med..

[23] Boel F., de Vos-Jakobs S., Riedstra N.S., Lindner C., Runhaar J., Bierma-Zeinstra S.M.A. (2024). Automated radiographic hip morphology measurements: an open-access method. Osteoarthritis Imaging.

[24] Wilkin G.P., Ibrahim M.M., Smit K.M., Beaulé P.E. (2017). A contemporary definition of hip dysplasia and structural instability: toward a comprehensive classification for acetabular dysplasia. J. Arthroplasty.

[25] Tannast M., Hanke M.S., Zheng G., Steppacher S.D., Siebenrock K.A. (2015). What are the radiographic reference values for acetabular under-and overcoverage?. Clin. Orthop. Relat. Res..

[26] Reijman M., Hazes J., Pols H., Koes B.W., Bierma-Zeinstra S. (2005). Acetabular dysplasia predicts incident osteoarthritis of the hip: the Rotterdam study. Arthritis Rheum..

[27] Thomas G., Palmer A., Batra R.N., Kiran A., Hart D., Spector T. (2014). Subclinical deformities of the hip are significant predictors of radiographic osteoarthritis and joint replacement in women. A 20 year longitudinal cohort study. Osteoarthritis Cartilage.

[28] Harris J.D., Gerrie B.J., Varner K.E., Lintner D.M., McCulloch P.C. (2016). Radiographic prevalence of dysplasia, cam, and pincer deformities in elite ballet. Am. J. Sports Med..

[29] Nötzli H.P., Wyss T.F., Stoecklin C.H., Schmid M.R., Treiber K., Hodler J. (2002 May). The contour of the femoral head-neck junction as a predictor for the risk of anterior impingement. J Bone Joint Surg Br.

[30] van Buuren M., Arden N.K., Bierma-Zeinstra S., Bramer W.M., Casartelli N.C., Felson D.T. (2020). Statistical shape modeling of the hip and the association with hip osteoarthritis: a systematic review. Osteoarthritis Cartilage.

[31] Arevalo N., Santamaria N., Diez E., Gredilla Molinero J., Grande Barez M. (2016).

[32] Lindner C., Thiagarajah S., Wilkinson J.M., Wallis G.A., Cootes T.F., arcOGEN Consortium (2013). Fully automatic segmentation of the proximal femur using random forest regression voting. IEEE Trans. Med. Imag..

[33] Nicholls A.S., Kiran A., Pollard T.C.B., Hart D.J., Arden C.P.A., Spector T. (2011 Nov). The association between hip morphology parameters and nineteen-year risk of end-stage osteoarthritis of the hip: a nested case-control study. Arthritis Rheum..

[34] Gamer M., Lemon J., Gamer M.M., Robinson A., Kendall’s W. (2012). Package ‘irr’.

[35] Nelson A.E., Stiller J.L., Shi X.A., Leyland K.M., Renner J.B., Schwartz T.A. (2016). Measures of hip morphology are related to development of worsening radiographic hip osteoarthritis over 6 to 13 year follow-up: the Johnston County Osteoarthritis Project.

[36] Yang W., Ye Q., Ming S., Hu X., Jiang Z., Shen Q. (2020 Nov). Feasibility of automatic measurements of hip joints based on pelvic radiography and a deep learning algorithm. Eur. J. Radiol..

[37] Tontanahal S., Madhuri V. (2021). Reproducibility of radiographic measurements made in the active stages of legg-calvé-perthes disease: evaluation of a prognostic indicator and an interim outcome measure. J. Pediatr. Orthop..

[38] Schwarz G.M., Simon S., Mitterer J.A., Huber S., Frank B.J., Aichmair A. (2023). Can an artificial intelligence powered software reliably assess pelvic radiographs?. Int. Orthop..

[39] Powell J., Gibly R.F., Faulk L.W., Carry P., Mayer S.W., Selberg C.M. (2020 Jul). Can EOS imaging substitute for conventional radiography in measurement of acetabular morphology in the young dysplastic hip?. J. Pediatr. Orthop..

[40] Engesæter I.Ø., Laborie L.B., Lehmann T.G., Sera F., Fevang J., Pedersen D. (2012). Radiological findings for hip dysplasia at skeletal maturity. Validation of digital and manual measurement techniques. Skeletal Radiol..

[41] Air M.E., Harrison J.R., Nguyen J.T., Kelly B.T., Bogner E.A., Moley P.J. (2019 Feb). Correlation of measurements of the prearthritic hip between plain radiography and computed tomography. Pharm. Manag. PM R.

[42] Lerch S., Kasperczyk A., Berndt T., Rühmann O. (2016 Oct). Ultrasound is as reliable as plain radiographs in the diagnosis of cam-type femoroacetabular impingement. Arch. Orthop. Trauma Surg..

[43] Mast N.H., Impellizzeri F., Keller S., Leunig M. (2011 Jan). Reliability and agreement of measures used in radiographic evaluation of the adult hip. Clin. Orthop. Relat. Res..

[44] Faber B.G., Ebsim R., Saunders F.R., Frysz M., Smith G.D., Cootes T. (2021).

[45] Tannast M., Hanke M.S., Zheng G., Steppacher S.D., Siebenrock K.A. (2015).

[46] Tannast M., Siebenrock K.A., Anderson S.E. (2007). Femoroacetabular impingement: radiographic diagnosis—what the radiologist should know. Am. J. Roentgenol..

