# Supplementary material for: Reliability and agreement of manual and automated morphological radiographic hip measurements
Source: Osteoarthr Cartil Open. 2024 Aug 14;6(3):100510. doi: 10.1016/j.ocarto.2024.100510 (PMC11387701; doi:10.1016/j.ocarto.2024.100510)
Supplement: Multimedia component 1 [file mmc1.pdf]

Supplement 1: Protocol for landmark annotation

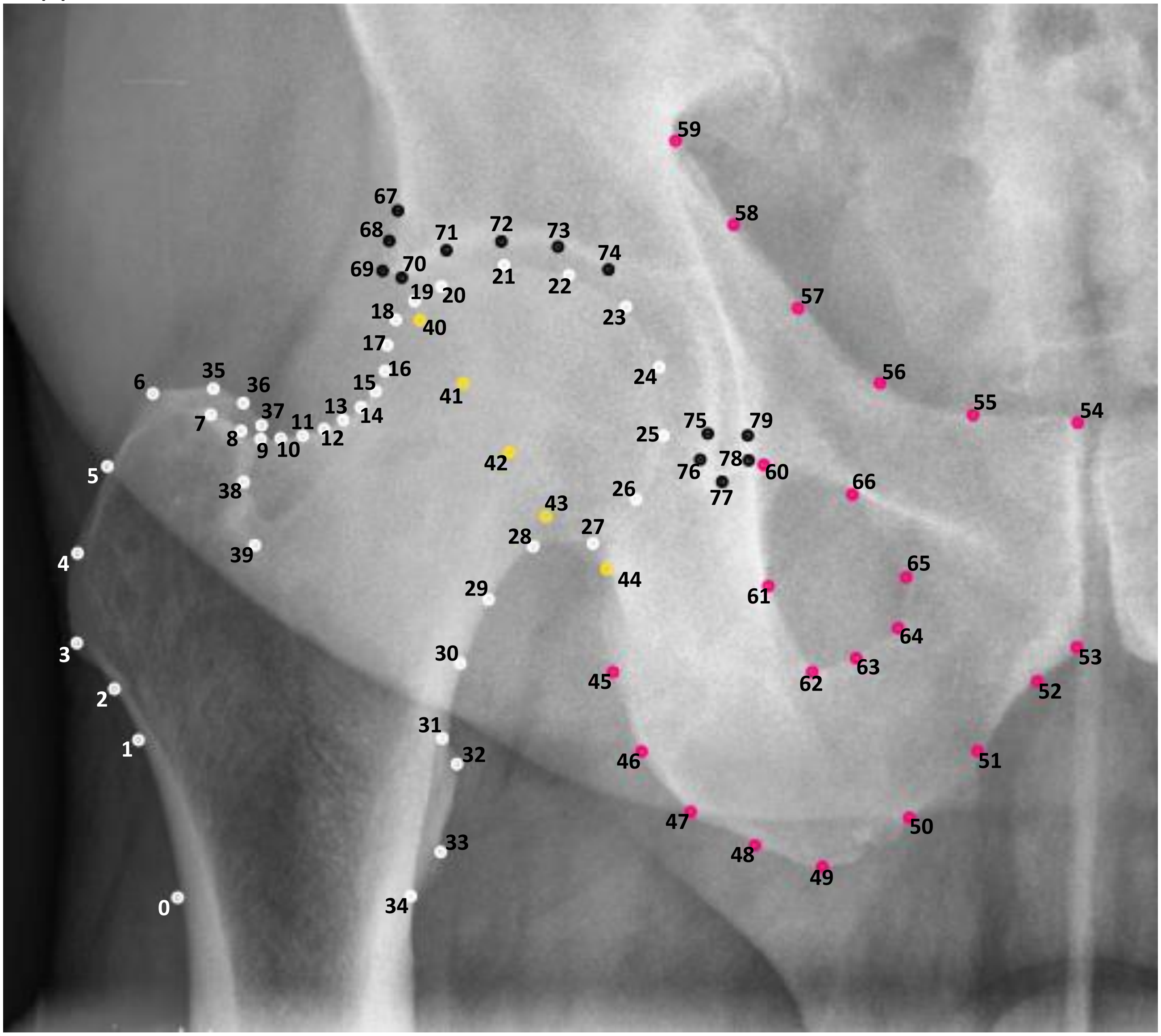

<u>Proximal femur (white points)</u>

<u>Lesser trochanter</u>

Point (**34**): Where the lesser trochanter starts bending off the shaft distally. If the lesser trochanter is seen behind the shaft, place this point on the cortex of the shaft at this level. If the lesser trochanter is not visible at all: missing points.

Point (**31**): Where the lesser trochanter joins the shaft proximally. If the lesser trochanter is seen behind the shaft, place this point on the cortex of the shaft at this level. If the lesser trochanter isn't visible at all: missing points.

Point (**32**)+(**33**): Respectively on the lower and upper corners of the lesser trochanter. If there are no clear corners: space them equally between (31) and (34) along the bony contour of the lesser trochanter.

Rest of proximal femur
Point (**0**) + (**1**): Respectively across (34) and (31) on the lateral femoral shaft. If point (1) would be above point (3) based on the position of point (34), place point (1) just under point (3).
Point (**3**): On the lower lateral corner of the greater trochanter.
Point (**2**): Equally spaced between (1) and (3).
Point (**6**): On the upper lateral corner of the (anterior) greater trochanter.
Point (**4**)+(**5**): Equally spaced between (3) and (6).
Point (**7**): On the medial upper corner of the anterior greater trochanter. If not visible, place this point equally spaced between (6) and (8) on the contour of the anterior greater trochanter.
Point (**8**): Where the anterior greater trochanter intersects the femoral.
Point (**18**): On the superolateral side of the femoral head, where the "best fitting circle" around the convexity of the femoral head seems to start. In case of a cam bump, osteophyte, or other irregularity: place (18) right after this bump ends, and the circle begins.
Point (**27**): On the inferomedial side of the femoral head, where the convexity of the femoral head seems to end. (The neck bends off after this point).
Point (**20-26**): Place these points equally spaced between (18) and (27) following the femoral head contour, unless there is a clear fovea dip, in which case the adjacent points, usually (24) and (25), are placed just outside of the fovea. Point (23) will be approximately placed halfway across the 'semi'-circle between (18) and (27).
Point (**9-17**): Place these points equally spaced between (8) and (18) following the lateral femoral neck contour. In case of irregularities like a cam bump or osteophyte, follow the outlining contour as closely as possible.
Point (**19**): Place this point equally spaced between (18) and (20) on the femoral head contour.
Point (**28**): At the deepest point of the inferomedial concavity of the femoral neck, so that (27-31) will follow the medial cortex of the femoral neck as closely as possible.
Point (**29**)+(**30**): Place these points equally spaced between (28) and (31), following the medial cortex of the femoral neck.

Greater trochanter, posterior part
** If the posterior greater trochanter is not visible: (35-39) missing points.
Point (**36**): On the upper medial corner of the posterior greater trochanter.
Point (**35**): Between (6) and (36), following the contour. If there is a clear corner, put it there.
Point (**37**): On the medial corner of the posterior greater trochanter, where it starts to drop downwards (caudal). This is independent of the femoral neck, so it can be before or after it dips behind the femoral neck, depending on the rotation of the proximal femur.
Point (**38**): Where the posterior greater trochanter is dropping straight down, right before it bends medially.
Point (**39**): On the end of the sclerotic line right *after* the medial bend, following the contour of the posterior greater trochanter.

Posterior wall of acetabulum (yellow points)
Point (**40**): On the uppermost visible part of the posterior wall of the acetabulum (usually right below the lateral edge of the weight-bearing surface or lateral osteophyte/pincer).

Point (**44**): Where the posterior wall joins the ischium (where the ischium usually proceeds vertically down).
Point (**41**-**43**): Place these points equally spaced between (40) and (44), following the contour of the posterior wall of the acetabulum.

<u>Ischium & Pubis (pink points)</u>
Point (**49**): On the most caudal point of the ischium (ischial tuberosity). If the ischial tuberosity appears as a straight line, put it in the middle of the ischial tuberosity.
Point (**45-48**): Place these points equally spaced between (44) and (49) along the contour of the ischial tuberosity.
Point (**52**): In the concavity before the symphysis.
Point (**50**)+(**51**): Place these points equally spaced between (49) and (52), following the caudal contour of the inferior pubic ramus.
Point (**53**): On the most caudal point of the pubic symphysis.
Point (**54**): On the most cranial point of the pubic symphysis.
Point (**59**): On the iliopectineal line of the pelvis, at the height where the ilioischial line splits off.
Point (**55-58**): Place these points equally spaced between (54) and (59). Follow the iliopectineal line, ignoring the ischial spine.
Point (**60**): In the superolateral corner of the obturator foramen.
Point (**62**): In the inferolateral corner of the obturator foramen.
Point (**61**): Equally spaced between (60) and (62), following the contour of the lateral rim of the obturator foramen.
Point (**64**): In the inferomedial corner of the obturator foramen.
Point (**63**): Place this point equally spaced between (62) and (64), following the contour/angle of the inferior rim of the obturator foramen.
Point (**65**): In the superomedial corner of the obturator foramen.
Point (**66**): Place this point equally spaced between (65) and (60), following the contour/angle of the superior rim of the obturator foramen.

<u>Acetabulum (black points)</u>
<u>Acetabular roof</u>
*** Points (70-74) along the weight-bearing zone (sourcil) are placed on the inferior rim of the sclerotic line.*
Point (**69**): On the most lateral point of the acetabulum, this can also be a lip/osteophyte.
Point (**70**): On the most lateral point of the **weight-bearing zone** (sourcil) of the acetabulum (most lateral point of sclerotic line).
Point (**74**): On the most medial point of the weight-bearing zone (sourcil) of the acetabulum, this is also the most superolateral point of the acetabular fossa. Usually there is a clear angle in the (sclerotic) line at the transition of weight-bearing zone to fossa. If the acetabular fossa is not visible at all, just place it on the most medial point of the sclerotic line.
Point (**71-73**): Along the underside of the sourcil, place these points equally spaced between (70) and (74), following the contour of the weight-bearing zone

Point (**68**): On the 'dimple' above (70), where the acetabular lip contour has a bend. When the acetabular lip forms a straight line, equally space point (68) and (67) above point (69), with the same distance as points (71-72).

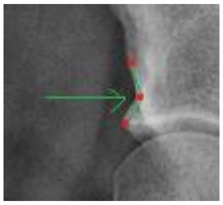

Point (**67**): Above (68), following the most lateral sclerotic line, with a similar distance between points (67-68) as points (71-72).

<u>*Pelvic teardrop*</u>
Point (**75**): On the superolateral corner of the visible teardrop (on the wall of the acetabular fossa)
Point (**77**): On the most caudal point of the teardrop.
Point (**79**): Across (75) on the other side of the teardrop.
Point (**76**)+(**78**): Across each other between (75-77-79), at the corners of the teardrop, where the more vertical (diverging) lines change direction to more oblique (converging) lines. This can be a very acute angle or more gradual.

Curve model:
Proximal femur curve: 0-1-2-3-4-5-6-7-8-9-10-11-12-13-14-15-16-17-18-19-20-21-22-23-24-25-26-27-28-29-30-31-32-33-34
Greater trochanter curve: 6-35-36-37-38-39
Posterior wall curve: 40-41-42-43-44
Ischium & pubis curve: 44-45-46-47-48-49-50-51-52-53-54-55-56-57-58-59
Foramen curve: 60-61-62-63-64-65-66
Acetabular roof curve: 67-68-69-70-71-72-73-74
Pelvic teardrop curve: 75-76-77-78-79

General rules:
- Osteophytes of the femoral head are *included* in the model. Follow the *outermost* contour. We can later correct for these with the radiological assessment data.
- Non-identifiable landmarks: missing points (write in separate log file)
- Only follow clear bony structures, not projecting shadows.
- Every hip is different, so not all anatomical landmarks might be clearly visible in each radiograph. In case of systematic doubt or error: discuss!
