# Supplementary material for: Reliability and agreement of manual and automated morphological radiographic hip measurements
Source: Osteoarthr Cartil Open. 2024 Aug 14;6(3):100510. doi: 10.1016/j.ocarto.2024.100510 (PMC11387701; doi:10.1016/j.ocarto.2024.100510)
Supplement: Multimedia component 2 [file mmc2.pdf]

Supplement 2: Example of the images for qualitative assessment

Below are depicted the visualizations of the acetabular depth-width ratio measurements as performed by observer 1, observer 2 and the automated method which were presented to the musculoskeletal radiologist for qualitative assessment of the measurement.

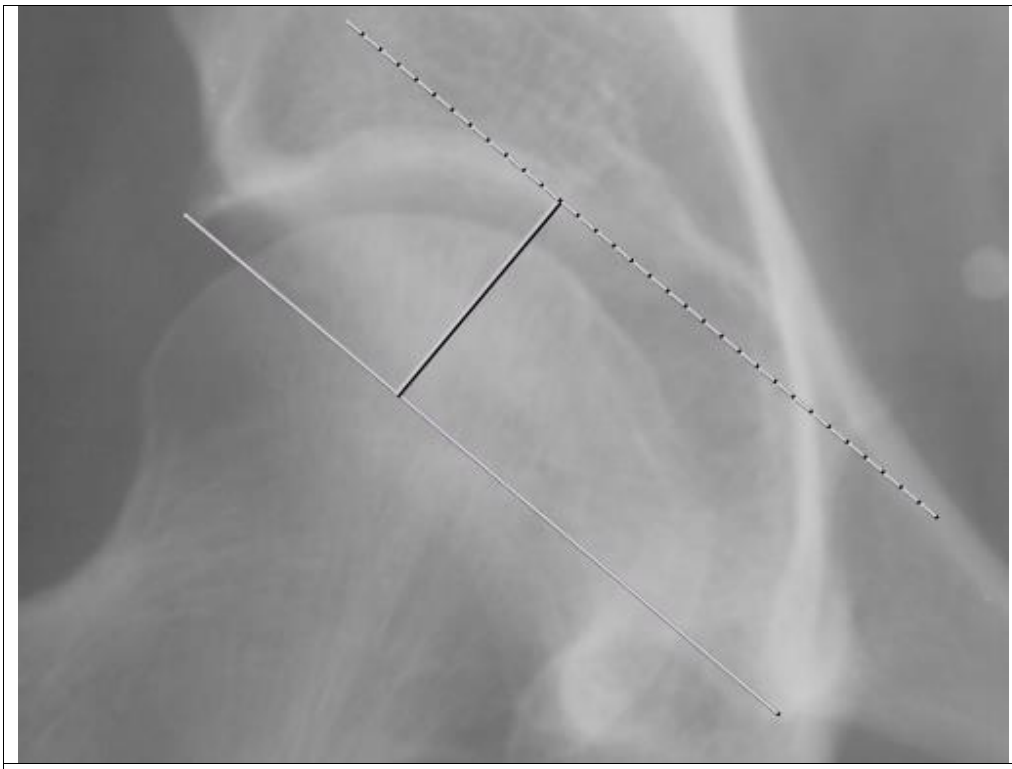

Visualization of the acetabular depth-width ratio measurement as performed by observer 1.

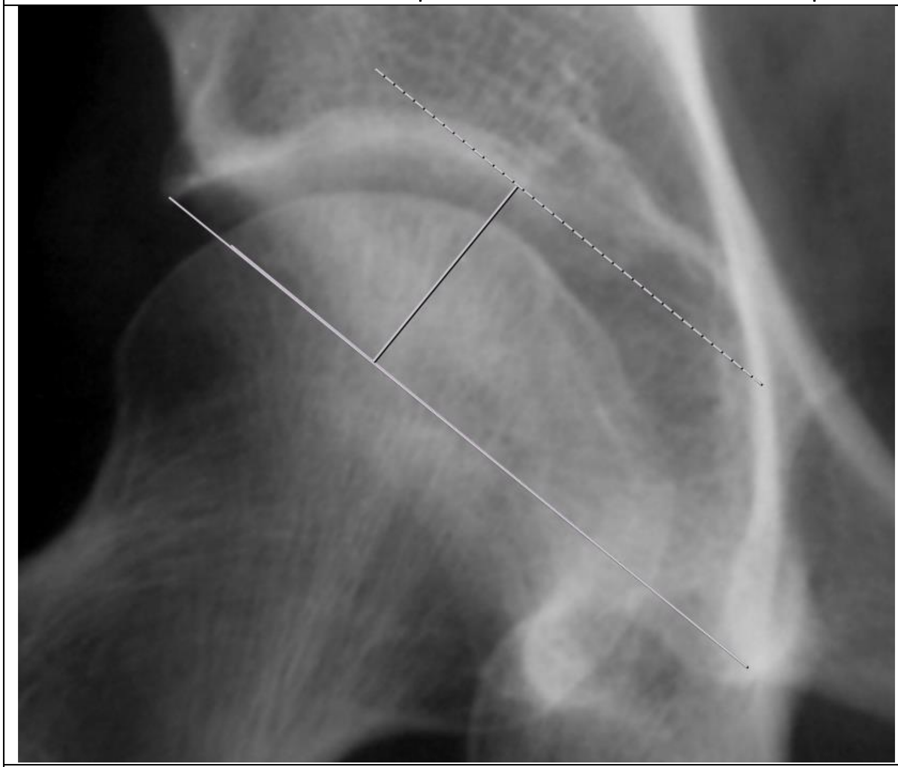

Visualization of the acetabular depth-width ratio measurement as performed by observer 2.

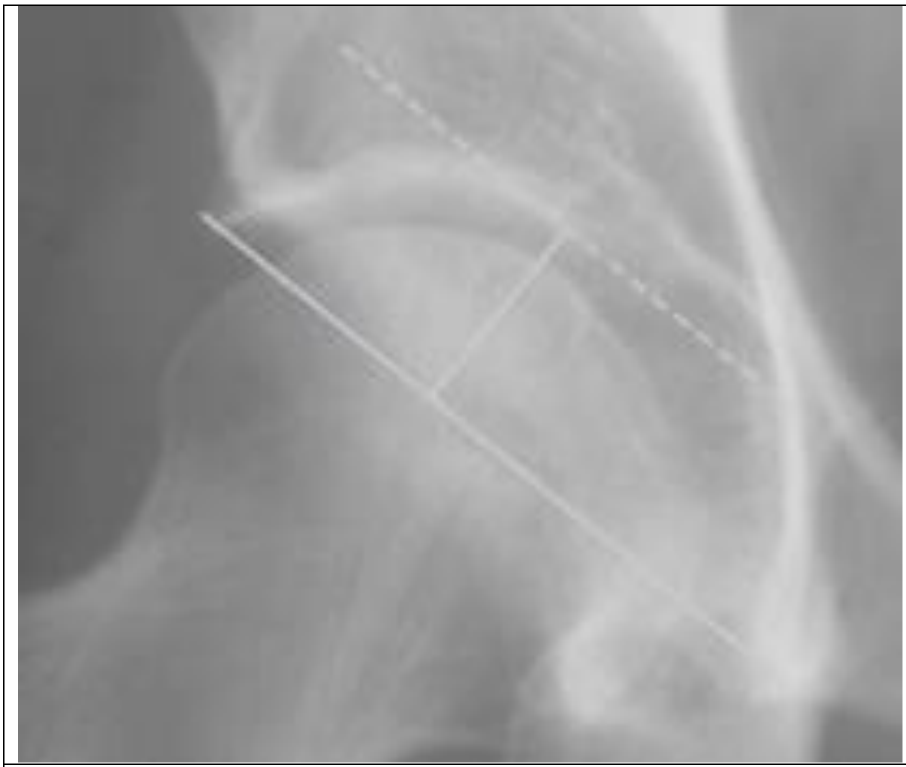

Visualization of the automated acetabular depth-width measurement on unadjusted landmark points.
